# Supplementary material for: Complex‐centric proteome profiling by SEC‐SWATH‐MS
Source: Mol Syst Biol. 2019 Jan 14;15(1):e8438. doi: 10.15252/msb.20188438 (PMC6346213; doi:10.15252/msb.20188438)
Supplement: Supplementary file 8 — Dataset EV7 [file MSB-15-e8438-s008.zip › feature_plots_string/Q9HCE7.pdf]

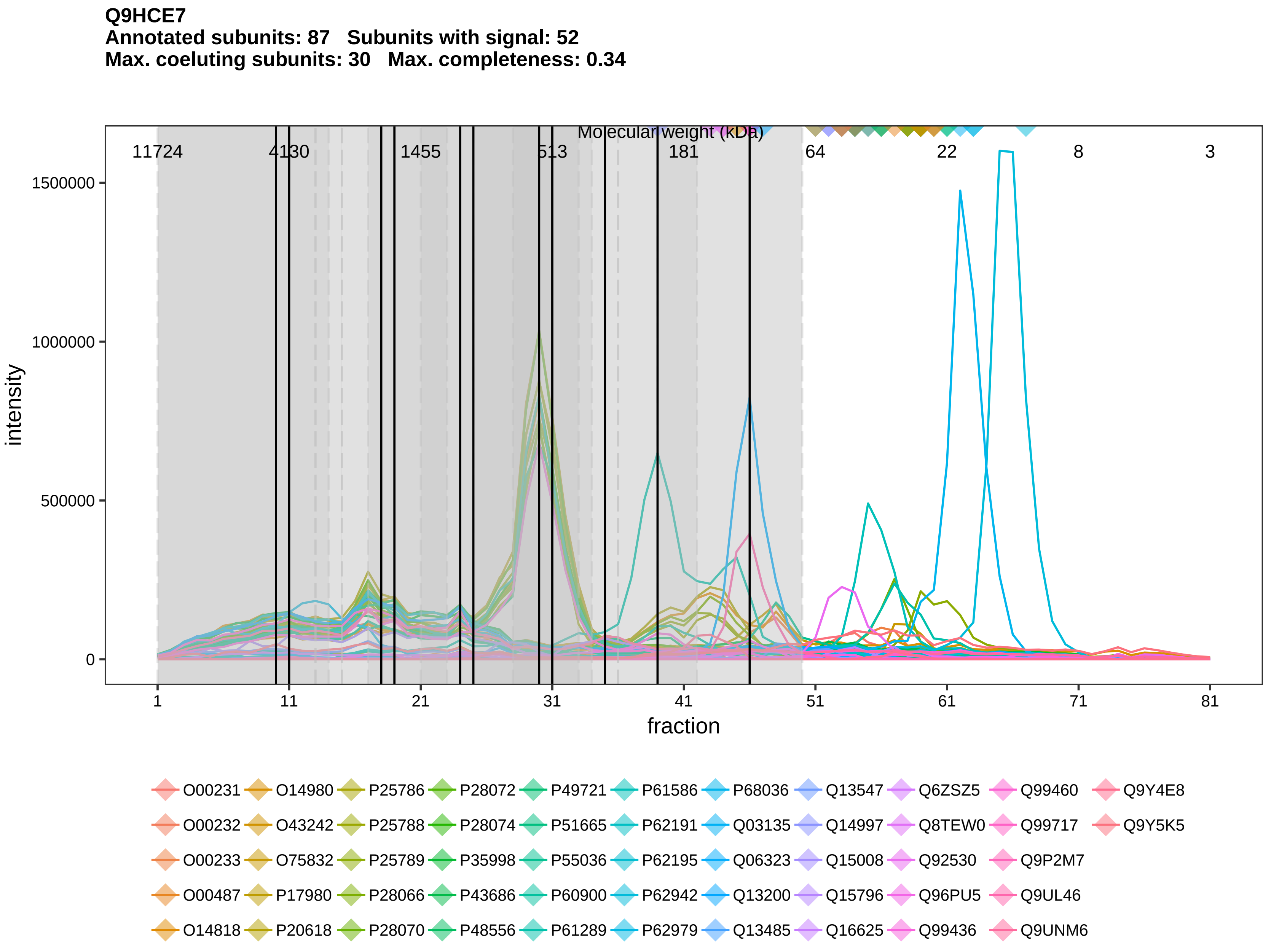

Q9HCE7
Annotated subunits: 87 Subunits with signal: 52
Max. coeluting subunits: 30 Max. completeness: 0.34
Molecular weight (kDa)
11724
4130
1455
513
181
64
22
8
3
intensity
1500000
1000000
500000
0
fraction
1
11
21
31
41
51
61
71
81
O00231
O00232
O00233
O00487
O14818
O14980
O43242
O75832
P17980
P20618
P25786
P25788
P25789
P28066
P28070
P28072
P28074
P35998
P43686
P48556
P49721
P51665
P55036
P60900
P61289
P61586
P62191
P62195
P62942
P62979
P68036
Q03135
Q06323
Q13200
Q13485
Q13547
Q14997
Q15008
Q15796
Q16625
Q6ZSZ5
Q8TEW0
Q92530
Q96PU5
Q99436
Q99460
Q99717
Q9P2M7
Q9UL46
Q9UNM6
Q9Y4E8
Q9Y5K5
